# Automated transperineal ultrasound analysis using deep learning for pelvic floor dysfunction assessment after total hysterectomy

**DOI:** 10.3389/fonc.2026.1730276

**Published:** 2026-06-18

**Authors:** Yanqing Xu, Fan Yang, Fan Zhao, Runyan Ji

**Affiliations:** 1 Ultrasonography Department, Affiliated Nantong Hospital 3 of Nantong University, Nantong Third People’s Hospital, Nantong, China; 2 School of Electrical Engineering and Automation, Nantong University, Nantong, China; 3 Medical School of Nantong University, Nantong University, Nantong, China

**Keywords:** deep learning, pelvic floor dysfunction (PFD), total hysterectomy, transformer, transperineal ultrasound (TPUS)

## Abstract

**Background:**

Pelvic floor dysfunction (PFD) is a common functional disorder following total hysterectomy. Currently, the assessment mainly relies on manual measurements of transperineal ultrasound (TPUS) images, which suffers from low efficiency, subjectivity, and limited reproducibility.

**Objective:**

To address these limitations, this work proposes an automated transperineal ultrasound analysis using deep learning for pelvic floor dysfunction assessment after total hysterectomy.

**Methods:**

A labeled dataset including key anatomical landmarks such as the symphysis pubis, bladder neck, urethra, and puborectalis muscle was established, and a multi-scale shifted window Transformer was developed to achieve automatic segmentation and key point detection. Additionally, a geometric reasoning module was further designed to compute seven clinically relevant functional parameters, including bladder neck–symphysis distance, posterior urethrovesical angle, and urethral rotation angle.

**Results:**

Experimental results demonstrated that the model achieved an average Dice coefficient of 88.67 ± 1.96% in segmentation, with key point localization errors controlled within 2 mm. The automatic measurement results are highly consistent with manual annotations, with Pearson correlation coefficients up to 0.92, and effectively distinguished functional differences among patients undergoing different surgical approaches.

**Conclusion:**

The proposed method enables structured, automated, and objective TPUS image analysis, significantly reducing manual intervention. Although validated in patients with benign diseases, the approach is directly transferable to gynecologic oncology patients, who are at even higher risk of PFD due to more extensive surgery and adjuvant therapies. It provides a reliable tool for postoperative functional monitoring and therapeutic evaluation after total hysterectomy, and holds great potential for functional imaging-based rehabilitation assessment in post-hysterectomy patients.

## 1 Introduction

Pelvic floor dysfunction (PFD) is a prevalent functional disorder among middle-aged and elderly women (1), primarily manifested as stress urinary incontinence, pelvic organ prolapse, and defecation disorders, all of which significantly impair quality of life. In recent years, with the increasing prevalence of gynecological surgeries such as hysterectomy, the incidence of PFD has markedly risen in postoperative populations (2, 3). Particularly after total hysterectomy, patients frequently experience varying degrees of pelvic floor muscle relaxation, reduced tissue elasticity, and degeneration of supportive structures. Without timely recognition and intervention, these functional impairments may progress to irreversible pelvic floor damage. Therefore, establishing an objective, quantitative, and efficient approach for pelvic floor function assessment is of great clinical significance for postoperative rehabilitation management, risk stratification, and long-term follow-up of patients after hysterectomy (4, 5). While this study focuses on patients with benign diseases to establish the methodology, the tool is equally applicable to gynecologic oncology patients, who often undergo more extensive surgery and adjuvant treatments that further increase the risk of PFD. Thus, the developed method holds direct translational relevance for both benign and malignant diseases.

Transperineal Ultrasound (TPUS) is a non-invasive, real-time imaging technique that has become a key tool in the evaluation of female pelvic floor anatomy (6–8). TPUS enables clear visualization of key anatomical landmarks, including the symphysis pubis (SP), bladder (BL), urethra (U), vagina (V), anus (A), rectum (R), and pelvic floor muscle (PR). By comparing images obtained at resting and during the Valsalva maneuver, TPUS allows the extraction of essential structural parameters, such as bladder neck-symphyseal distance (BSD), posterior urethrovesical angle (PUA), anorectal junction–symphysis distance (ASD), the anteroposterior diameter of the hiatus (HAPD), bladder neck descent (BND), urethral rotation angle (URA), and anorectal junction descent (ARJD) (9, 10). These seven parameters are internationally recognized clinical indicators of pelvic floor support and mobility, and are routinely used in clinical practice to evaluate PFD. These quantitative indicators are widely applied in postoperative rehabilitation assessment and monitoring of pelvic floor functional changes, providing substantial clinical value.

However, TPUS images are often characterized by blurred tissue boundaries, low signal-to-noise ratio, and substantial structural variability (11, 12), which restricts current clinical evaluation to visual interpretation and manual measurement by ultrasound technicians. This approach is not only inefficient and poorly reproducible but is also highly dependent on operator expertise and subjective judgment, making it difficult to support standardized large-scale, multicenter follow-up studies in patients after total hysterectomy (13, 14).

With the rapid development of artificial intelligence, particularly deep learning techniques in medical image analysis, automatic recognition and structural modeling of TPUS images have gradually become a research hotspot (15–18). Early studies, represented by convolutional neural networks (CNNs) such as U-Net (19), achieved remarkable progress in tissue segmentation tasks on grayscale ultrasound images. The encoder–decoder architecture of CNNs enables the effective recovery of local tissue boundaries. Nevertheless, due to the limited receptive field of CNNs, they have limitations in capturing long-range dependencies between anatomical structures (20, 21). More recently, Transformer-based models have been increasingly applied to medical imaging owing to their global attention mechanisms and superior contextual modeling capabilities. Compared with traditional CNNs, Transformers can effectively integrate global semantic information from low-contrast ultrasound images, thereby enabling accurate recognition and modeling of complex anatomical structures. They have demonstrated remarkable potential in tasks such as tumor detection, tissue segmentation, and structural modeling (22, 23). The development of this method marks a significant shift in approach, moving from convolutional networks driven by local feature extraction to self-attention models with global perceptual features, providing a completely new technical path for the automated and standardized analysis of TPUS images. Recent work by (24) proposed automatic extraction of the minimal hiatal plane from 3D pelvic floor ultrasound. In contrast, our method analyzes 2D TPUS images using a multi-scale shifted window Transformer for fully automated segmentation and key point detection, combined with a geometric reasoning module to compute seven functional parameters across both resting and Valsalva states. This enables comprehensive, quantitative assessment of postoperative pelvic floor function in benign cases, without manual plane selection.

To address the challenges of weak anatomical features, large regional variability, and blurred boundaries in TPUS images, this work proposes a segmentation network based on a multi-scale shifted window Transformer, combined with a key point detection module and a geometric reasoning module, to achieve fully automated identification and computation of seven pelvic floor functional parameters (BSD, PUA, URA, HAPD, ASD, ARJD, and BND). The research framework includes four main steps: (1) constructing a TPUS anatomical dataset with pixel-level structural annotations and key point heat map labels; (2) designing a segmentation network that integrates multi-scale shifted window attention, and introducing a structure-guided key point detection module; (3) developing a geometric reasoning module to automatically calculate seven functional parameters based on segmentation and key point predictions; and (4) validating the stability and clinical utility of the proposed model through accuracy evaluation, reproducibility analysis, and comparative studies across surgical groups of post-hysterectomy patients.

The proposed automated transperineal ultrasound analysis using deep learning has the potential to replace conventional manual delineation and measurement procedures, enabling standardized, objective, and automated assessment of pelvic floor function. This approach provides an efficient and scalable solution for postoperative rehabilitation monitoring and clinical decision support in patients after total hysterectomy.

## 2 Materials and methods

### 2.1 Subjects

This retrospective study was conducted following the STROBE guidelines and approved by the Ethics Committee of Nantong Third People’s Hospital (Approval No. EK2024081). All participants signed written consent forms after being informed of the details. All study procedures have been conducted in compliance with the Declaration of Helsinki.

A total of 92 female patients scheduled for total hysterectomy due to benign gynecological conditions between January 2020 and May 2025 were initially screened. Based on image quality and completeness of follow-up, 50 cases were finally selected for inclusion. The surgical indications include multiple uterine fibroids, uterine adenomyosis, cervical intraepithelial neoplasia, endometrial polyps, and refractory functional uterine bleeding, etc. The age of participants ranged from 35 to 65 years, and none had a history of severe pelvic infection or confirmed pelvic floor disorders preoperatively.

Before enrollment, comprehensive demographic and clinical data were systematically collected, including age, body mass index (BMI), obstetric history, menopausal status, history of difficult delivery, as well as risk factors such as chronic cough and constipation. All questionnaires were completed under the supervision of trained research staff.

Inclusion criteria were: (1) age between 30 and 80 years with normal cognitive ability; (2) number of deliveries ≤ 3 and more than 10 years after delivery; (3) ability to correctly perform a standardized Valsalva maneuver. Exclusion criteria included: (1) BMI >28 kg/m^2^; (2) comorbidities associated with sustained increases in intra-abdominal pressure, such as chronic cough or defecation disorders; (3) significant cardiopulmonary insufficiency, diabetes, or a history of heavy smoking; (4) history of pelvic radiotherapy, severe pelvic adhesions, or ongoing pelvic floor rehabilitation therapy; (5) history of twin pregnancy or macrosomia delivery; (6) inability to perform a proper Valsalva maneuver or poor image quality due to patient positioning.

**Figure 1:**
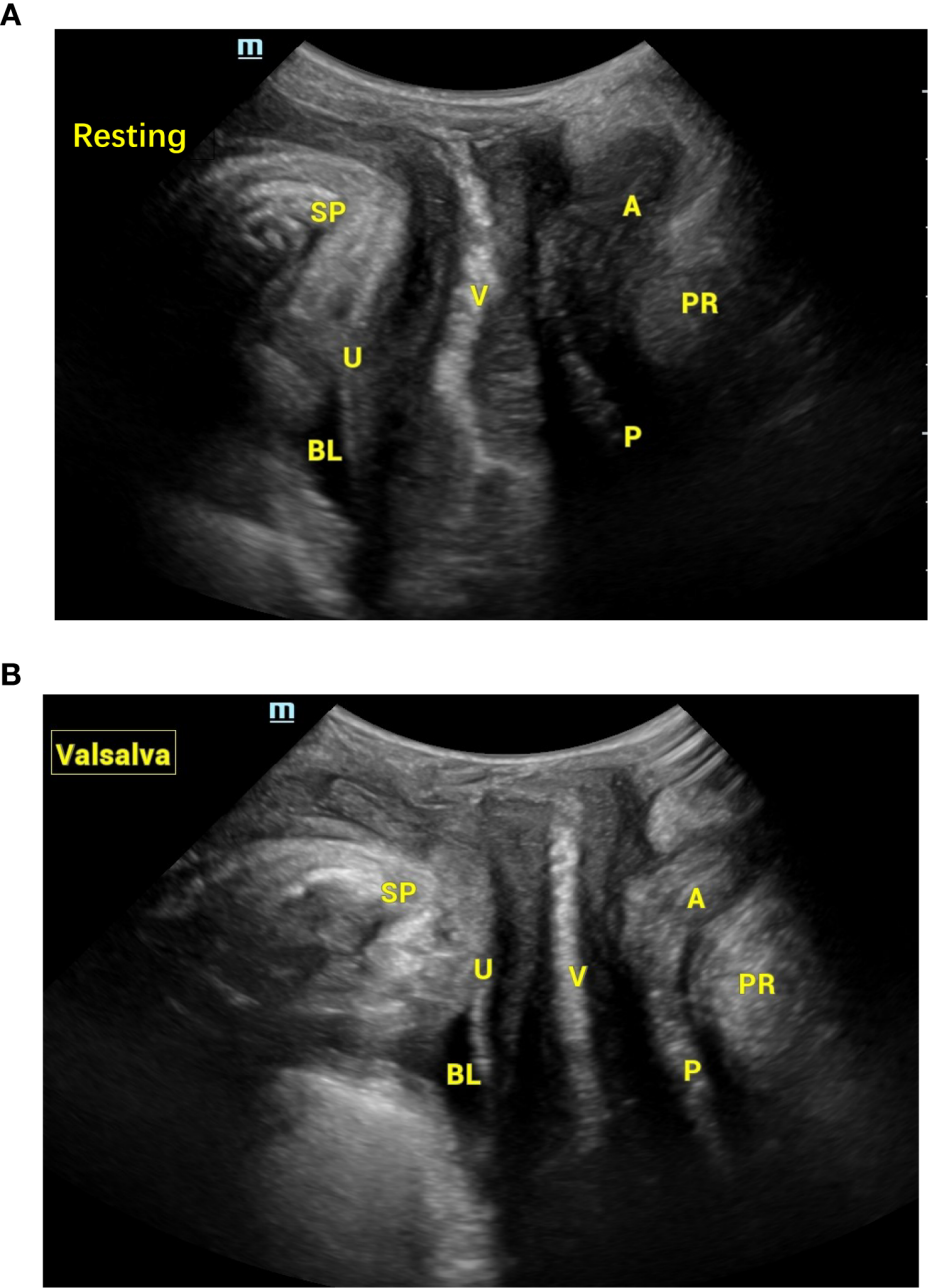
Representative midsagittal TPUS images. **(A)** Typical resting-state image showing SP, BL, U, and PR. **(B)** Image during Valsalva maneuver illustrating bladder neck descent and urethral rotation.

All examinations were performed using a Mindray RESONA R9T system with a C5–1 convex array probe (frequency range: 1–5 MHz). In the supine position with knees flexed, midsagittal transperineal images were acquired. TPUS gray-scale dynamic images (cine loop) were captured in both the resting state and the Valsalva maneuver state to ensure clear visualization of key anatomical landmarks such as SP, BL and U. **Figure 1** illustrates representative midsagittal TPUS images in the resting state and the Valsalva states. To ensure standardized assessment, Valsalva images showing maximal pelvic floor relaxation were selected for analysis, excluding frames with co-activation or suboptimal relaxation.

For each participant, dynamic cine loops of at least 30 frames were acquired. Image quality was required to meet the following criteria: (1) complete and clearly defined visualization of key anatomical structures; (2) absence of significant artifacts or motion-induced distortions; (3) standardized participant positioning and adherence to established acquisition protocols.

### 2.2 Data preprocessing

To enable automated recognition of pelvic floor anatomical structures and the measurement of functional parameters in TPUS images, all data were subjected to standardized preprocessing and annotation prior to input into the deep learning model, ensuring sample quality and consistency.

Firstly, from each dynamic cine loop sequence, the 10 frames with the highest image quality scores were selected based on a quantitative metric, as defined in Equation 1:

(1) $$Score=0.5\cdot Laplacian_{variance}+0.3\cdot Contrast_{norm}+0.2\cdot SNR_{norm},$$

where *Laplacian_variance_*is the normalized variance of the Laplacian of the image, *Contrast_norm_*is the normalized Michelson contrast, and *SNR_norm_*is the normalized signal-to-noise ratio. Each component is normalized to [0,1] across all frames of the same loop before summation. This metric prioritizes frames that clearly displayed key anatomical landmarks, including SP, BL, U and PR. Subsequently, a fixed rectangular region of interest (ROI) of size 300×250 pixels, which encompass all these anatomical areas was defined on the first frame by an experienced sonographer. The same ROI coordinates were applied uniformly to all frames from the same patient. The ROI was cropped to exclude irrelevant background and emphasize structural focus for the model (25).

All ROI images were then resized to 224×224 pixels and normalized to balance variations in brightness and contrast across images. To enhance model robustness against imaging noise and individual anatomical variability, several data augmentation strategies were applied, including histogram equalization, random rotation, mirror flipping, and Gaussian blurring, thereby improving generalization performance (26, 27).

Finally, two experienced attending physicians with more than 10 years of experience in pelvic floor ultrasonography performed frame-by-frame manual annotations of the SP, BL, U, and PR regions in the TPUS images to generate pixel-level segmentation labels (ground truth masks). In addition, Gaussian distributed heat maps were generated at the centroid of each anatomical structure to provide supervisory signals for key point detection tasks. All annotations underwent cross-checked and consistency-reviewed to ensure the accuracy and reliability of the labels.

### 2.3 Methods

To achieve automated recognition of key pelvic floor anatomical structures and standardized measurement of functional parameters in TPUS images, this work proposes a multi-scale shifted window Transformer (MSWT). The architecture is specifically designed to integrate the strengths of local texture perception and global anatomical dependency modeling, making it particularly well-suited for TPUS images characterized by blurred tissue boundaries, low contrast, and significant morphological changes.

**Figure 2:**
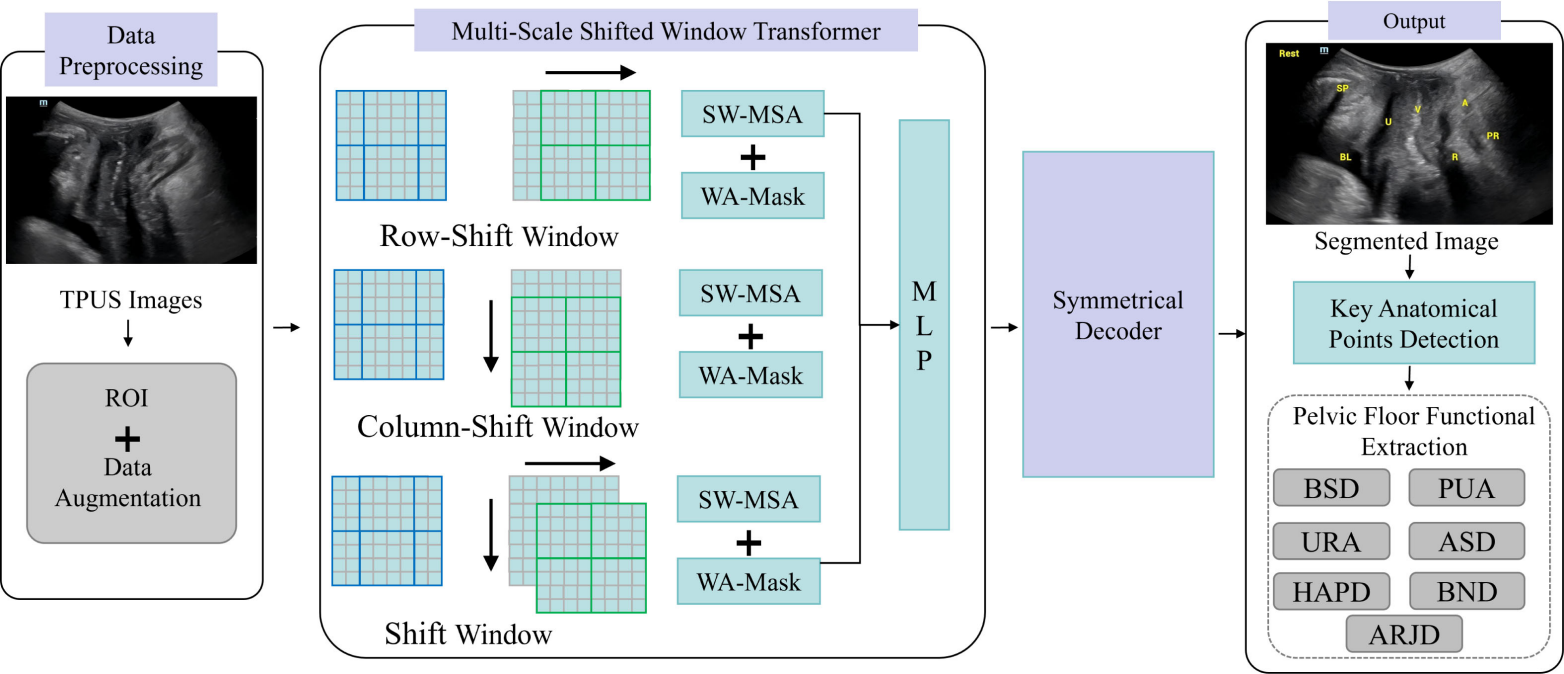
The overall framework of automated transperineal ultrasound analysis using deep learning. The framework consists of three main modules: a multi-scale shifted window Transformer (MSWT) for local and global feature extraction, a keypoint detection module that generates probability heatmaps of anatomical landmarks, and a geometric reasoning module for calculating pelvic floor functional parameters.

**Figure 2** illustrates the overall framework of automated transperineal ultrasound analysis using deep learning. The model comprises three major components, including an image segmentation backbone, a structural key point detection module, and a geometric reasoning module, enabling inference from pixel-level segmentation to the estimation of seven functional parameters. Specifically, the input TPUS images are first processed through a multi-scale shifted window Transformer backbone, incorporating shifted window-based multi-head self-attention (SW-MSA) and window-based attention masks (WA-Mask) to extract hierarchical texture and anatomical features. Subsequently, the key point detection module performs precise localization of SP, BL, and PR. Finally, the geometric reasoning module calculates pelvic floor functional parameters based on the spatial relationships among the detected key points.

#### 2.3.1 Multi-scale shifted window transformer

TPUS images are characterized by blurred boundaries, high levels of noise, and irregular structural morphology. Although CNNs demonstrate strengths in local texture recognition, their limited receptive fields restrict their ability to capture long-range dependencies among anatomical structures such as SP, BL, and PR (28). To address this limitation, the backbone network incorporates a multi-scale shifted window Transformer, which is built upon the Swin Transformer architecture. Our implementation is adapted from the official Swin Transformer repository, which can be available at https://github.com/microsoft/Swin-Transformer, and modified for multi-scale feature aggregation and window-based attention masking, as detailed below. In this architecture, SW-MSA is applied within each window to learn local semantic features (29). In addition, a shifted window strategy is introduced within the hierarchical architecture to facilitate cross-window information exchange (30), thereby enhancing global structural perception. Compared with existing self-attention frameworks that have been successfully applied to multi-scale spatiotemporal intent recognition in human–robot interaction (31, 32), our MSWT specifically adapts the multi-scale window partitioning mechanism to the challenging anatomical modeling task in TPUS, ensuring that anatomical structures at different scales can be effectively captured.

Furthermore, to increase the model’s sensitivity to key anatomical regions, WA-Mask (33) is employed, assigning higher weights to key points during attention computation. Specifically, the proposed WA-Mask biases the attention scores toward window regions that contain key anatomical landmarks, including SP, BL and PR.

First, a spatial saliency map $S\in {\mathbb{R}}^{H\times W}$ is generated the features of the deepest encoder through a lightweight 1 × 1 convolution layer, where each pixel value represents the probability of belonging to anatomically important regions. Then, for each window *w* at scale *s*, the average saliency score within the window is calculated as follows Equation 2:

(2) $${\bar{s}}_{w}=\frac{1}{\left|w\right|}\sum_{i\in w}S_{i},$$

where |w| denotes the number of pixels in window *w*. The corresponding window attention mask is subsequently defined as Equation 3:

(3) $$M_{w}=\gamma \cdot {\bar{s}}_{w},$$

where *γ* is an empirically determined amplification coefficient and is set to 2.0 in this work. Windows containing anatomically relevant structures will receive larger attention responses during the feature interaction process.

For each scale, we compute the WA-Mask independently for all windows at that scale, and apply the mask within the self-attention computation at that scale. The masks at different scales respectively guide the network to focus on anatomical regions that are most relevant to the corresponding scale.

During self-attention computation, the generated WA-Mask is added to the attention probability values before the softmax operation, as shown in Equation 4:

(4) $$\text{Attention}\left(Q,K,V\right)=\text{Softmax}\left(\frac{QK^{T}}{\sqrt{d}}+M\right)V,$$

where *Q*, *K*, and *V* denote the query, key, and value matrices, respectively, and *M* contains the mask values of all windows. By enhancing attention responses in anatomically informative regions, the proposed WA-Mask improves structural awareness and boundary continuity under the TPUS conditions with noise and low contrast. This design enables the network to simultaneously capture local edge textures and global structural relationships, substantially improving the stability of boundary recognition in TPUS images compared with traditional CNNs.

#### 2.3.2 Key anatomical point detection module

**Figure 3:**
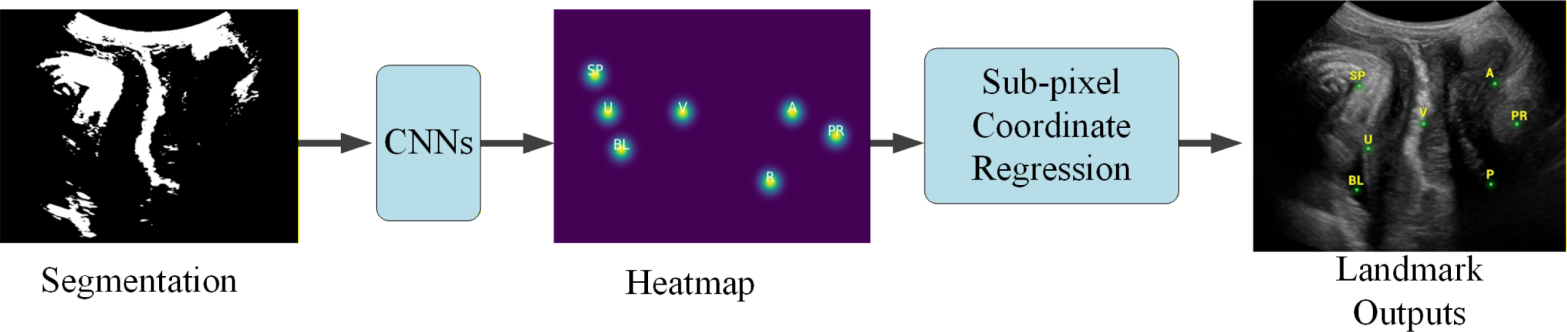
Workflow of key anatomical point detection module. CNNs generate Gaussian heatmaps from segmentation features. Coarse landmarks are located via argmax, refined by quadratic surface fitting on 3 × 3 patches, and evaluated using Euclidean distance.

Building upon the segmentation feature maps, a structural key point detection module was constructed to perform heatmap regression and generate probability maps of key anatomical landmarks, such as SP, BL, and PR, as shown in **Figure 3**.

During training, ground-truth heatmaps are generated by placing a 2D Gaussian kernel at each annotated landmark coordinate, as defined in Equation 5:

(5) $$H_{k}^{*}\left(x,y\right)=\text{exp }\left(-\frac{{\left(x-x_{k}^{*}\right)}^{2}+{\left(y-y_{k}^{*}\right)}^{2}}{2\sigma^{2}}\right),$$

where $\left(x_{k}^{*},y_{k}^{*}\right)$ denotes the ground-truth coordinate, and *σ* controls the spatial spread of the Gaussian kernel, which is set to 3 pixels. This formulation encourages the network to learn spatial confidence distributions centered at anatomical landmarks.

During inference, the landmark positions are determined via an argmax operation on the predicted heatmap. To achieve sub-pixel accuracy, a quadratic surface is fitted to the 3 × 3 neighborhood around each peak, and the refined coordinates are derived by locating the maximum of the fitted surface (34). This sub-pixel refinement effectively alleviates localization errors caused by blurred boundaries and low contrast in ultrasound images.

The final predicted coordinate $\left(x_{pred},y_{pred}\right)$ is then converted to physical units using DICOM pixel spacing. Localization accuracy is quantified by the Euclidean distance between predicted and ground-truth coordinates (*x_gt_*, *y_gt_*), as shown in Equation 6:

(6) $$d=\sqrt{{\left(x_{pred}-x_{gt}\right)}^{2}+{\left(y_{pred}-y_{gt}\right)}^{2}}.$$

The obtained key point coordinates were subsequently employed as geometric inputs for pelvic floor functional parameter computation, providing a robust foundation for millimeter-level precision and standardized parameter outputs, thereby supporting quantitative evaluation and dynamic analysis of postoperative pelvic floor function.

#### 2.3.3 Geometric reasoning module

The geometric reasoning module automatically computes seven pelvic floor functional parameters based on the key point coordinates of TPUS, namely BSD, PUA, URA, ASD, HAPD, BND, and ARJD. Specifically, BSD is obtained as the Euclidean distance between BL and SP; PUA and URA are calculated based on the angle formed by the vectors of key points, reflecting the spatial direction relationship between the urethra and the bladder neck. ASD, HAPD, and BND are inferred from longitudinal (head-totail direction) displacements and anteroposterior distances of relevant keypoints, thereby characterizing morphological changes in pelvic floor soft tissues and cavities. ARJD represents the distance between anatomical structures associated with the anorectal junction (ARJ). All parameter calculations are performed with precise pixel-to-physical length conversion based on DICOM metadata, enabling millimeter-level calibration. This ensures high consistency between automated measurements and manual annotations, while conforming to clinical standardization requirements, thus providing a reliable basis for quantitative evaluation of postoperative pelvic floor function.

#### 2.3.4 Multi-task joint loss function

To achieve simultaneous optimization of anatomical region segmentation and key point localization, a multi-task joint loss function was designed during training. Building upon the preceding feature extraction and network design, this composite objective enables the network to learn both spatially coherent anatomical boundaries and anatomically meaningful keypoint positions in a unified framework. Specifically, the segmentation task incorporates both Dice loss and cross-entropy loss to jointly ensure regional completeness and boundary sharpness, while the key point detection branch employs a mean squared error (MSE) loss to supervise heatmap regression. The overall objective is formulated as Equation 7:

(7) $${ℒ}_{total}={\text{λ}}_{1}{ℒ}_{seg}+{\text{λ}}_{2}{ℒ}_{kp}+{\text{λ}}_{3}{ℒ}_{other},$$

where λ_1_, λ_2_, and λ_3_ denote task-specific weighting coefficients, which are empirically tuned on the validation set to balance segmentation accuracy and localization precision.

The segmentation loss ${ℒ}_{seg}$ integrates Dice loss and cross-entropy loss as.


$${ℒ}_{seg}=\alpha L_{\text{dice}}+\left(1-\alpha \right)L_{\text{ce}},$$

where *α* is a balancing factor that adjusts the contribution of each component.

Dice loss measures the regional overlap between the predicted probability map *P_i_* and the corresponding ground truth mask *G_i_*, as defined in Equation 8:

(8) $$L_{\text{dice}}=1-\frac{2\sum_{i=1}^{N}P_{i}G_{i}}{\sum_{i=1}^{N}P_{i}^{2}+\sum_{i=1}^{N}G_{i}^{2}},$$

where *N* denotes the total number of pixels, *P_i_* ∈ [0,1] is the predicted probability of pixel *i* belonging to the target region, and *G_i_* ∈ {0,1} represents the corresponding binary label.

The cross-entropy loss evaluates pixel-wise classification consistency as shown in Equation 9.

(9) $$L_{\text{ce}}=-\frac{1}{N}\sum_{i=1}^{N}\left[G_{i}\text{log }(P_{i})+\left(1-G_{i}\right)\text{log }(1-P_{i})\right],$$

penalizing deviations between predicted and true class probabilities.

For key point detection, the MSE loss quantifies the pixel-wise discrepancy between predicted and ground-truth heatmaps, as defined in Equation 10:

(10) $$L_{\text{kp}}=\frac{1}{K}\sum_{k=1}^{K}\sum_{i=1}^{N}{\left({\hat{H}}_{i}\left(k\right)-H_{i}\left(k\right)\right)}^{2},$$

where *K* denotes the number of keypoints, and ${\hat{H}}_{i}\left(k\right)$ and *H_i_*(*k*) represent the predicted and ground-truth heatmap intensities of the *k*-th keypoint at pixel *i*, respectively.

In addition, ${ℒ}_{other}$ represents auxiliary regularization terms that constrain the network parameters and stabilize training. These may include an *L*_2_ weight decay term to prevent overfitting by penalizing excessively large model parameters, and a smoothness regularization term to enforce spatial continuity in the predicted probability maps, as shown in Equation 11:

(11) $${ℒ}_{other}=\beta_{1}{∥\theta ∥}_{2}^{2}+\beta_{2}\sum_{i=1}^{N}{∥\nabla P_{i}∥}_{2}^{2},$$

where *θ* denotes the trainable network parameters, ∇*P_i_* is the spatial gradient of the predicted probability at pixel *i*, and *β*_1_, *β*_2_ are hyperparameters controlling the regularization strength.

By jointly optimizing segmentation, detection, and regularization objectives, the proposed loss function enables the network to achieve anatomically consistent segmentation with sub-pixel key point precision while maintaining model stability and generalization across diverse ultrasound imaging conditions.

#### 2.3.5 Model training and implementation

To improve robustness against imaging variability and anatomical differences, extensive data augmentation was applied, including random rotation, horizontal/vertical flipping, brightness and contrast adjustments, and Gaussian blurring. Specifically, random rotation within ±10^°^, scaling 0.9–1.1, horizontal flipping with probability 0.5, and intensity jitter (brightness ±0.2, contrast ±0.2). The random seed for all augmentation operations was fixed at 42, and this value is also used as the seed for other operations to ensure the reproducibility of the results.

A total of 50 TPUS cases were included, resulting in 1500 image frames with corresponding annotations. The dataset was partitioned at the patient level to prevent data leakage, with 35 patients and 1050 frames used for training, 7 patients and 225 frames for validation, and 8 patients and 225 frames for testing, as summarized in **Table 1**.

**Table 1:** TPUS dataset partition for model training and evaluation.

Dataset split	Number of patients	Number of frames	Percentage
Training	35	1050	70%
Validation	7	225	15%
Testing	8	225	15%
Total	50	1500	100%

The Adam optimizer was used with an initial learning rate of 1 × 10^−4^, batch size of 8, and 200 training epochs. Early stopping with a patience of 20 epochs and a ReduceLROnPlateau scheduler with a factor of 0.5 and patience of 10 epochs were employed to prevent overfitting and ensure stable convergence. The loss weight coefficients were empirically set based on the performance of the validation set, namely *λ*_1_ = 1.0, *λ*_2_ = 0.5, *λ*_3_ = 0.01, *α* = 0.5, *β*_1_ = 0.0001, and *β*_2_ = 0.001.

Model training and inference were implemented in PyTorch 1.12.1 on an NVIDIA RTX 3090 GPU (24 GB) and Intel Xeon CPU. Inference time per TPUS image was approximately 0.12 s, enabling near real-time clinical analysis. The complete source code will be publicly released at https://github.com/fany1993usst-coder/MSWTcode.

## 3 Results

To validate the performance advantages of the proposed multi-scale shifted window Transformer in TPUS image automatic recognition and functional parameter inference, this work conducted a systematic analysis and comparative evaluation from four aspects: segmentation accuracy, key point localization precision, functional parameter measurement consistency, and clinical discriminative capability.

### 3.1 General characteristics

A total of 50 patients who underwent total hysterectomy for benign lesions were enrolled in this work. General clinical characteristics of patients are summarized in **Table 2**. Among them, 28 patients received total abdominal hysterectomy (TAH), while 22 patients underwent total laparoscopic hysterectomy (TLH). No statistically significant differences were observed between the two groups in terms of age, BMI, gravidity, parity, or menopausal status (all *p* ≥ 0.05), indicating comparable baseline characteristics.

**Table 2:** General clinical characteristics of patients.

Indicator	TAH group (n = 28)	TLH group (n = 22)	t/χ2	p value
Age (years)	54.18 ± 6.21	52.95 ± 5.84	t = 0.821	0.416
BMI (kg/m)	24.92 ± 1.93	24.05 ± 1.88	t = 1.851	0.069
Gravidity	2.18 ± 0.92	2.27 ± 0.80	t = 0.398	0.693
Parity	1.42 ± 0.50	1.36 ± 0.49		0.686
Menopausal/	24/4	19/3	t = 0.407	0.962
non-menopausal			χ2 = 0.002	

### 3.2 Pelvic floor functional parameters

Based on predicted key point coordinates, seven structural parameters were automatically measured through a geometric reasoning module. Physical calibration was performed using the pixel-to-millimeter conversion factor from DICOM files to ensure consistency with manual measurements in both units and scale. The definitions of these parameters are as follows:

- Bladder Neck–Symphyseal Distance (BSD): Vertical distance from the bladder base to the inferior margin of the pubic symphysis, evaluating bladder support capacity.
- Posterior Urethrovesical Angle (PUA): Angle between the bladder neck and the urethral axis, reflecting the elasticity of posterior urethral support structures.
- Anorectal Junction–Symphyseal Distance (ASD): Linear distance from the inferior margin of the pubic symphysis to the anorectal junction (ARJ).
- Anteroposterior Diameter of the Hiatus (HAPD): Anteroposterior diameter of the levator hiatus, measured from the inferior margin of the pubic symphysis to the anterior margin of the puborectalis muscle under Resting and Valsalva.
- Bladder Neck Descent (BND): Inferior displacement of the bladder neck under Valsalva, a key clinical index for bladder neck function.
- Urethral Rotation Angle (URA): Rotation of the urethra under Valsalva relative to Resting, reflecting urethral mobility.
- Anorectal Junction Descent (ARJD): Inferior displacement of the ARJ under Valsalva, commonly used to assess perineal mobility.

### 3.3 Segmentation performance evaluation

For TPUS segmentation task, Dice similarity coefficient (DSC), Intersection over Union (IoU), and segmentation accuracy (Accuracy) were employed to quantify agreement between model predictions and expert manual annotations. DSC measures overlap between predicted and true regions, IoU evaluates joint coverage and boundary precision, and Accuracy calculates the proportion of correctly classified pixels to complement DSC and IoU. These metrics provide a comprehensive assessment of model performance on low-contrast TPUS images.

As shown in **Table 3**, the proposed multi-scale shifted window Transformer outperformed the classical U-Net, Attention U-Net (35), and Swin-Unet (36) in the TPUS segmentation task. The average Dice coefficient reached 88.67 ± 1.96%, representing an improvement of 3.22% compared with Swin-Unet. This indicates that the model demonstrates superior contour recognition and boundary restoration capabilities for key anatomical structures such as SP, BL, and PR in TPUS images characterized by low contrast and blurred boundaries.

**Table 3:** Comparison of segmentation performance of different models on TPUS images.

Model	Dice (%)	IoU (%)	Accuracy (%)
U-Net	82.14 ± 2.87	71.53 ± 3.12	90.83 ± 2.37
Attention U-Net	84.92 ± 2.43	74.36 ± 2.79	91.87 ± 2.05
Swin-Unet	85.45 ± 2.12	76.21 ± 2.54	92.63 ± 1.92
Proposed method	88.67 ± 1.96	79.83 ± 2.11	94.21 ± 1.54

Compared with the baseline model, U-Net tended to mis-segment regions with blurred boundaries, leading to the lowest Dice scores. Attention U-Net improved the recognition of local structures by introducing attention mechanisms, but it still exhibited limitations in long-range feature interactions. Swin-Unet enhanced overall segmentation accuracy through local-global feature modeling, but remained insufficient in boundary refinement of critical anatomical structures.

The proposed method utilizes a multi-scale shifted window mechanism to facilitate cross-regional information exchange and integrates a WA-Mask to assign greater attention weights to critical anatomical structures, including the bladder neck, PR, and ARJ. Consequently, enhanced segmentation accuracy and consistency were achieved in these key regions compared with the baseline models.

To further assess the stability and robustness of the proposed framework under different data partitions, five-fold cross-validation was conducted at the patient level. Specifically, the dataset was randomly divided into five mutually exclusive subsets, where four folds were used for training and the remaining fold was used for validation in each iteration. The final cross-validation Dice was reported as the mean ± standard deviation across the 5 folds. Detailed fold-wise segmentation results are summarized in **Table 4**.

**Table 4:** Five-fold cross-validation results for TPUS image segmentation.

Fold	Dice (%)	IoU (%)	Accuracy (%)
1	87.12	78.21	93.81
2	86.85	77.94	93.52
3	88.23	79.56	94.21
4	84.29	75.03	92.43
5	90.05	81.76	95.02
Mean ± SD	87.31 ± 2.23	78.50 ± 2.56	93.80 ± 1.0

The average Dice coefficient across all folds was 87.31 ± 2.23%, closely matching the held-out test set performance of 88.67 ± 1.96%. The small difference is expected due to variations in train-test splits and limited patient numbers per fold. These results indicate that the proposed model maintains stable segmentation performance across different splits, providing initial evidence of internal generalizability, though external validation on multi-center, multi-device datasets remains necessary.

### 3.4 Ablation study

To systematically evaluate the contributions of each component in the proposed architecture, we conducted an ablation study on the validation set. Five model variants were designed:

- w/o Multi-scale: The multi-scale module is removed to assess its contribution to capturing features at different resolutions.
- w/o WA-Mask: The weighted attention mask is removed to evaluate the effect of attention-guided feature refinement.
- w/o Weighted Loss: The weighted loss is replaced with a standard unweighted loss to quantify the effect of loss weighting.
- w/o Data Augmentation: All data augmentation strategies (e.g., flipping, rotation, scaling) are removed to evaluate their impact on model generalization and stability.
- w/o Post-processing: Post-processing refinements, including sub-pixel keypoint fitting, are disabled to evaluate their contribution to final prediction accuracy and boundary refinement.

The ablation results in **Table 5** demonstrate that each component contributes to overall performance. Removing the multi-scale module caused the largest decrease in Dice and IoU, highlighting the importance of multi-scale feature extraction. WA-Mask and weighted loss contributed moderate improvements, showing their role in feature refinement and loss optimization. Data augmentation significantly enhanced model generalization and stability, while post-processing refinements improved boundary accuracy and prediction quality. The full model achieved the best performance across all metrics, confirming the effectiveness of the proposed architecture.

**Table 5:** Ablation study.

Variants	Dice (%)	IoU (%)	Accuracy (%)
w/o Multi-scale	85.94 ± 2.31	76.58 ± 2.67	92.84 ± 1.88
w/o WA-Mask	86.72 ± 2.18	77.41 ± 2.43	93.26 ± 1.76
w/o Weighted Loss	86.35 ± 2.24	77.03 ± 2.51	93.11 ± 1.82
w/o Data Augmentation	85.47 ± 2.42	76.12 ± 2.73	92.57 ± 1.95
w/o Post-processing	87.81 ± 2.07	78.94 ± 2.26	93.78 ± 1.63
Full Model (MSWT)	88.67 ± 1.96	79.83 ± 2.11	94.21 ± 1.54

### 3.5 Evaluation of key point localization accuracy

For key anatomical point detection, Euclidean distance was used to evaluate positional deviation. The mean error, standard deviation (SD), maximum error, and accuracy within 3 mm (%) between the predicted coordinates and the manually annotated coordinates were calculated to comprehensively assess localization performance. These metrics intuitively reflect the model’s positioning accuracy for the bladder neck, pubic symphysis, midline of the urethra, and puborectalis muscle, thereby providing a reliable foundation for subsequent geometric parameter inference.

The evaluation results, as shown in **Table 6**, showed that the proposed model achieved low localization errors for most anatomical structures in the resting state. Specifically, the mean localization errors of SP and BL were 1.72 mm and 1.64 mm, respectively, both below 2 mm, with localization accuracy (≤3 mm) exceeding 95%. The overall mean error across all landmarks was 1.94 ± 0.65 mm, corresponding to a 93.8% accuracy, demonstrating high precision and robustness in static conditions.

**Table 6:** Accuracy evaluation of TPUS anatomical landmark localization in resting and Valsalva states.

Key structure	Mean error (mm)	SD (mm)	Max error (mm)	Accuracy ≤ 3 mm (%)	State
SP	1.72	0.59	2.81	95.2	resting
	1.96	0.63	3.10	94.1	Valsalva
BL	1.64	0.55	2.70	97.0	resting
	1.88	0.60	2.92	95.6	Valsalva
UM	1.82	0.61	2.91	94.8	resting
	2.04	0.68	3.18	92.4	Valsalva
V	1.95	0.64	3.05	93.7	resting
	2.15	0.71	3.26	92.1	Valsalva
A	2.01	0.69	3.22	93.0	resting
	2.17	0.71	3.41	91.8	Valsalva
R	2.04	0.70	3.35	92.7	resting
	2.20	0.74	3.45	91.3	Valsalva
PR	2.07	0.72	3.37	92.5	resting
	2.23	0.75	3.48	91.1	Valsalva
Overall	1.94 ± 0.65	–	–	93.8	resting
	2.12 ± 0.68	–	–	92.7	Valsalva

During the Valsalva maneuver, the mean localization errors of all landmarks slightly increased, with the overall error rising from 1.94 ± 0.65 mm to 2.12 ± 0.68 mm, while the accuracy within 3 mm decreased marginally from 93.8% to 92.7%. This increase primarily results from the displacement and morphological deformation of pelvic floor structures caused by the Valsalva maneuver, which increases localization difficulty. More noticeable error increments were observed for UM, V, and PR, reflecting the dynamic complexity and elasticity of these structures under load-bearing conditions. Nevertheless, the localization accuracy remained clinically acceptable (mean accuracy of 92.7%), confirming that the proposed method can robustly capture anatomical landmarks under both resting and straining states, thereby supporting reliable quantitative assessment of postoperative pelvic floor function and dynamic parameter extraction.

**Figure 4:**
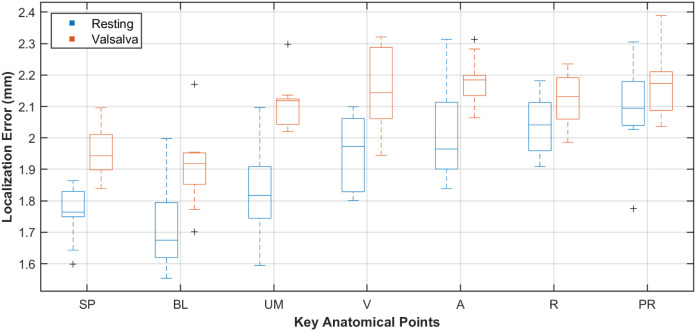
Boxplots of localization errors of TPUS key anatomical points in resting and Valsalva states.

**Figure 4** illustrates the boxplots of localization errors for TPUS key anatomical points in both resting and Valsalva states. In the resting state, the errors are tightly clustered, with median values generally below 2 mm, indicating stable and precise localization. In contrast, during the Valsalva maneuver, the errors exhibit a slightly wider dispersion and higher median values, particularly for UM, V, and PR. These variations reflect the morphological deformation and positional shifts of pelvic floor structures caused by the maneuver. This visualization intuitively demonstrates the error distribution across different anatomical landmarks and functional states, providing a clear basis for understanding localization behavior and supporting dynamic parameter extraction under functional loading. Despite the slight increase in errors during Valsalva, the overall localization accuracy remained within the clinically acceptable threshold (≤ 3 mm), confirming the robustness and adaptability of the proposed model in both static and dynamic conditions.

Furthermore, comparison between the resting and Valsalva states facilitates the extraction of dynamic functional parameters, such as bladder neck descent, urethral rotation angle variation, and elasticity changes in pelvic floor muscles. These parameters contribute to a more comprehensive functional evaluation and provide valuable clinical insights for assessing postoperative recovery and monitoring pelvic floor dysfunction.

In summary, the proposed multi-scale window attention Transformer network achieved high accuracy, robustness, and clinical feasibility in automatic TPUS key point localization. By effectively modeling both static and dynamic pelvic floor features, the network establishes a reliable foundation for quantitative analysis of structural parameters and functional assessment in clinical practice.

### 3.6 Consistency of pelvic floor functional parameters

To evaluate agreement between automated and clinical manual measurements, mean absolute error (MAE) and root mean square error (RMSE) were calculated to quantify systematic bias and dispersion, respectively. Pearson correlation coefficient (*r*) was calculated between the automated measurements and the manual measurements for each functional parameter. A value of *r* ≥ 0.85 was considered indicative of good linear consistency. Additionally, the 95% limits of agreement (LoA) is used to determine whether the model has met the clinically acceptable consistency standard, and to measure the frequency with which the automatic measurement values fall within the acceptable range compared to the manual reference values. These statistical approaches are standard in method comparison studies for medical imaging and are widely adopted in recent ultrasound-based deep learning research (37). Seven pelvic floor functional parameters were selected as evaluation metrics: BSD, PUA, ASD, HAPD, BND, URA, and ARJD. Among these, BSD, PUA, ASD, and HAPD were measured under both resting and Valsalva conditions to evaluate the model’s adaptability to anatomical structures under stable and stressed states. BND, URA, and ARJD were derived from the difference between Resting and Valsalva states to reflect the dynamic activity of the pelvic floor.

To quantify the impact of keypoint localization errors on derived functional parameters, we conducted an error propagation analysis based on Monte Carlo simulation. Using the standard deviations of keypoint localization errors as input uncertainty, zero-mean Gaussian noise was added to the keypoint coordinates for each test frame with 1,000 perturbation iterations per frame. All functional parameters were recalculated for each perturbation, and the propagated uncertainty for each parameter was derived. For dynamic parameters such as BND, URA and ARJD, we have further defined an error amplification factor, which is the ratio of the propagation uncertainty of the dynamic parameter to the uncertainty of the corresponding static parameter (BSD or PUA). This reflects the cumulative uncertainty resulting from subtracting two independent measurements.

**Table 7** summarizes the automatic measurement errors, correlation analysis, and error propagation results. Among all functional parameters, R-BSD showed the highest agreement with manual measurements, with a Pearson correlation coefficient of 0.92, consistent with the maximum correlation value reported in the abstract. 95% LoA coverage all exceeded 90%, indicating high stability and reliability across different anatomical and functional metrics. For distance-based parameters (BSD, ASD, HAPD), MAE remained approximately 2 mm, consistent with prior deep learning-based TPUS studies, demonstrating that the model accurately captures spatial relationships and geometric features between key pelvic floor points.

**Table 7:** Automatic measurement errors and correlation analysis of pelvic floor functional parameters.

Parameter	MAE	RMSE	Pearson r	95% LoA coverage	Propagated uncertainty (SD)	Error magnification
R-BSD	1.58 mm	1.94 mm	0.92	93.5%	1.54 mm	1.00
V-BSD	1.66 mm	2.01 mm	0.90	92.8%	1.69 mm	1.10
R-PUA	3.54°	3.96°	0.88	92.3%	3.28°	1.00
V-PUA	3.71°	4.13°	0.86	92.0%	3.51°	1.07
R-ASD	1.82 mm	2.25 mm	0.87	91.5%	1.85 mm	1.00
V-ASD	1.93 mm	2.36 mm	0.86	90.9%	1.96 mm	1.00
R-HAPD	1.74 mm	2.09 mm	0.89	91.8%	1.78 mm	1.00
V-HAPD	1.83 mm	2.19 mm	0.88	91.3%	1.88 mm	1.00
BND	2.05 mm	2.46 mm	0.85	90.6%	2.28 mm	1.48
URA	3.82°	4.27°	0.85	91.0%	4.85°	1.48
ARJD	2.21 mm	2.62 mm	0.84	90.2%	2.36 mm	1.53

^1^ R-denotes Resting condition, V-denotes Valsalva condition. Error magnification for BND, URA, and ARJD is the ratio of the propagated uncertainty of the dynamic parameter to that of the corresponding static parameter (BSD or PUA). For ASD and HAPD, which are single-time-point distance parameters, no difference-related error amplification occurs, and the magnification factor is set to 1.00.

In contrast, dynamic parameters BND and URA showed slightly higher errors, approximately 2 mm or 3.8°, primarily due to rapid displacement and deformation of anatomical structures under the Valsalva maneuver, compounded by motion artifacts and boundary blurring. Error propagation analysis further quantified this observation. The propagated uncertainty of BND was 2.28 mm and that of URA was 4.85°, with corresponding error magnification factors of approximately 1.48–1.53, indicating that uncertainties from resting and Valsalva measurements accumulate, leading to larger measurement uncertainty for dynamic parameters. For ASD and HAPD, which are single-time-point distance parameters, the propagated uncertainties were 1.85–1.96 mm with error magnification factors close to 1.0. Angular parameters, such as PUA and URA, maintained errors within 3.5° to 4.3°, which remains clinically acceptable, and PUA exhibited high correlation under both states, with Pearson r values ranging from 0.86 to 0.88, indicating robust modeling of the relative geometry between the bladder and urethra.

In summary, the proposed automated method achieves comparable accuracy to manual annotation for static parameters and demonstrates good consistency for dynamic functional metrics. Considering the error ranges of MAE and RMSE, approximately 2 mm for distances and 4°for angles, satisfy clinical follow-up and preoperative assessment requirements, this method provides practical value by reducing manual operation dependence and minimizing subjective variability, offering a technical foundation for standardized quantitative evaluation of pelvic floor function.

### 3.7 Clinical discriminative ability assessment

At the clinical application level, the proposed model was further evaluated for its feasibility and discriminative capability in automated pelvic floor parameter computation. TPUS images from patients who underwent TAH and TLH were analyzed to compare postoperative pelvic floor functional indicators. Independent-sample *t*-tests were conducted to assess intergroup differences. To account for multiple comparisons without relying on parametric assumptions, a non-parametric permutation test was applied. Patient labels were randomly permuted 5000 times, and t statistics were recalculated for each pelvic floor parameter in each permutation. Parameters with adjusted *p* ≤ 0.05 were considered statistically significant. As shown in **Table 8**, only V-BSD showed a statistically significant difference, with an adjusted *p*-value of 0.042. The other dynamic parameters demonstrated moderate effect sizes, but due to the limited sample size, they failed to reach a significant level. These results indicate that the model can reliably extract parameters sensitive to surgical outcomes, and larger-scale studies are needed to establish its clinical significance.

**Table 8:** Comparison of pelvic floor functional parameters under Resting and Valsalva conditions between different surgical approaches.

Parameter	TAH (n=28)	TLH (n=22)	p-value (raw)	p-value (adjusted, permutation)	Cohen’s d	95% CI of difference
R-BSD (mm)	24.42 ± 2.59	25.51 ± 2.49	0.132	0.423	0.43	(-2.55, 0.37)
V-BSD (mm)	15.37 ± 3.06	17.84 ± 3.07	0.006	0.042	0.81	(0.80, 4.14)
R-PUA (°)	124.56 ±9.37	123.76 ±13.15	0.798	0.998	0.07	(-6.32, 5.42)
V-PUA (°)	142.68 ±8.34	136.77 ±11.65	0.047	0.412	0.58	(-11.67, -0.15)
R-ASD (mm)	16.72 ± 2.58	17.81 ± 3.24	0.183	0.611	0.37	(-2.10, 1.18)
V-ASD (mm)	11.34 ± 2.91	12.14 ± 3.31	0.360	0.879	0.26	(-1.78, 1.18)
R-HAPD (mm)	49.79 ± 2.10	48.75 ± 2.28	0.095	0.284	0.47	(-0.22, 2.30)
V-HAPD (mm)	54.03 ± 2.65	53.09 ± 3.42	0.267	0.724	0.31	(-0.82, 2.70)
BND (mm)	9.06 ± 2.29	7.67 ± 2.37	0.037	0.215	0.60	(0.09, 2.69)
URA (°)	30.82 ± 5.39	27.33 ± 6.41	0.037	0.227	0.59	(0.09, 5.89)
ARJD (mm)	9.20 ± 2.00	8.50 ± 2.20	0.180	0.579	0.33	(-0.66, 2.06)

^1^ R-denotes Resting condition, V-denotes Valsalva condition. BND, URA, and ARJD are dynamic parameters.

Notably, BND, as a key dynamic parameter reflecting bladder neck descent, sensitively captures postoperative weakening of pelvic floor support structures. Its elevation is closely associated with increased risk of urinary incontinence and cystocele. Similarly, abnormal variations in PUA and URA indicate reduced stability of the bladder urethra, suggesting potential voiding dysfunction or impaired muscular coordination in some patients. Although these functional changes may not immediately manifest as clinical symptoms, they could serve as important predictors of postoperative complications during long-term follow-up. In contrast, static anatomical parameters such as BSD, ASD, and HAPD showed no significant differences between the two groups, indicating that the two surgical approaches have relatively limited direct impact on pelvic floor morphology. This finding aligns with previous studies, which suggest that anatomical structures remain relatively stable at resting, whereas functional parameters under dynamic conditions better reflect surgical differences and potential functional impairments.

To assess whether the observed group differences are clinically meaningful, we benchmarked them against minimal clinically important difference (MCID) values reported in the pelvic floor ultrasound literature. For BND, a difference of approximately 5–8 mm is associated with clinically detectable changes in stress urinary incontinence severity. The observed mean BND difference between TAH and TLH was 1.39 mm, which is substantially below the MCID threshold. For URA, the group difference was 3.49°, whereas clinically relevant URA changes typically exceed 10°–15° when differentiating severity grades. The largest observed difference was V-BSD, but moderate measurement variability in BSD suggests that changes below 5 mm may not reliably indicate functional impairment. Therefore, while some parameters reached nominal statistical significance before correction, their magnitudes remain below established clinical thresholds, indicating that the surgical approach has relatively modest effects on postoperative pelvic floor function.

In summary, the automatically computed dynamic parameters demonstrate high sensitivity and stability, providing quantitative evidence for postoperative risk stratification and rehabilitation assessment. These results indicate that the model can reliably extract parameters sensitive to surgical outcomes, and larger-scale studies are needed to establish its clinical significance.

### 3.8 Comparison of segmentation result

**Figure 5:**
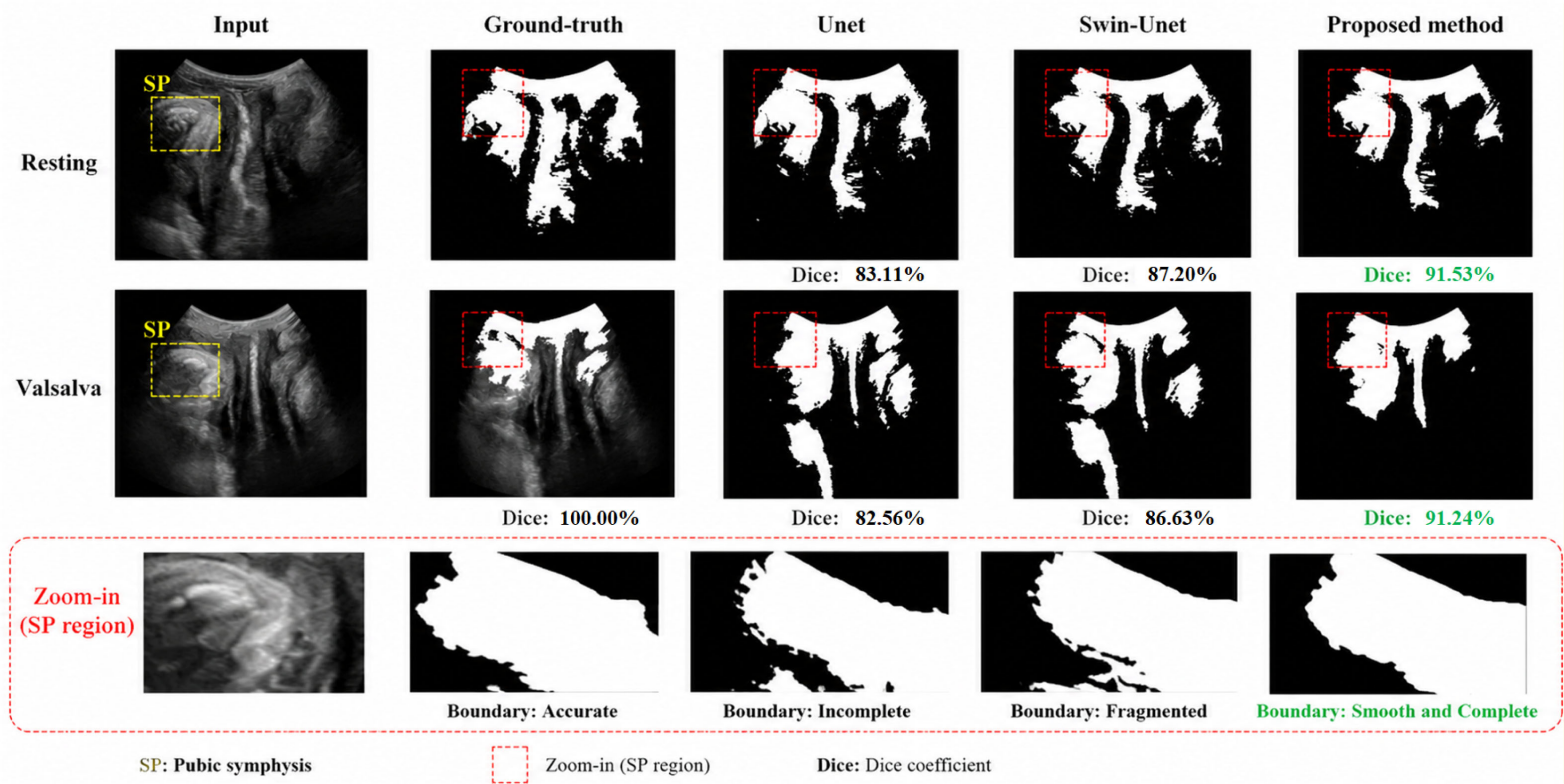
Comparison of segmentation results among different models.

To provide an intuitive evaluation of segmentation performance, visual comparisons among different models are presented in **Figure 5**. The figure includes the original ultrasound image, ground-truth annotation, segmentation results from U-Net, Swin-Unet, and the proposed model, as well as Dice coefficients indicated below each result. Zoom-in views of SP region are also provided to highlight boundary details.

As shown in the figure, U-Net produces relatively coarse segmentation results with noticeable fragmentation and incomplete boundaries, particularly in low-contrast regions, resulting in lower Dice coefficients (e.g., 83.11% and 82.56%). Swin-Unet improves the continuity of the structure by integrating global context information, yielding smoother and more coherent segmentation with moderately higher Dice (e.g., 87.20% and 86.63%), although slight over-smoothing and residual artifacts remain. In contrast, the proposed model achieves the clearest and most accurate segmentation. It effectively suppresses noise, preserves structural integrity, and generates smooth and complete boundaries, as evidenced by the highest Dice coefficients, such as 91.53% and 91.24%. Zoom-in views of the SP region further highlight the precise boundary delineation and improved segmentation continuity compared to baseline methods. This gradual improvement clearly demonstrates the advantage of global modeling based on transformers in capturing complex anatomical structures in ultrasound images.

## 4 Discussion

This work proposes a multi-scale shifted window Transformer, enabling fully automated identification of key pelvic floor anatomical structures and comprehensive functional parameter inference from TPUS images. Experimental results demonstrate that the proposed method achieves superior performance in segmentation accuracy, key point localization, functional parameter consistency, and clinical discriminative capability, validating its potential clinical utility for postoperative monitoring of PFD.

In terms of segmentation performance, the proposed multi-scale shifted window Transformer network significantly outperformed traditional convolutional architectures (U-Net, Attention U-Net) and single-scale Transformers (Swin-Unet), achieving a Dice coefficient of 88.67 ± 1.96%. This improvement is primarily attributed to SW-MSA, which enhances global semantic perception while modeling local structures, effectively mitigating common TPUS imaging challenges such as low contrast and blurred boundaries. Additionally, the integration of WA-Mask strengthened the model’s focus on critical anatomical regions, including the pubic symphysis, bladder neck, and anorectal junction, further improving the accuracy and stability of anatomical structure recognition.

Regarding key point localization, the bladder neck and pubic symphysis were identified with errors below 2 mm, within clinically acceptable thresholds. Although the anorectal junction exhibited slightly higher errors due to ultrasound signal attenuation and deep anatomical characteristics, overall errors remained below 2.1 mm. These findings are consistent with previous studies and indicate that accurate boundary recognition of deep structures remains a technical challenge in TPUS automated analysis, which could be further addressed using 3D-TPUS imaging or cross-modal image fusion techniques.

For functional parameter consistency, static parameters such as BSD and HAPD exhibited measurement errors below 2 mm, while angular parameters such as PUA and URA were controlled within 4°, with Pearson correlation coefficients exceeding 0.84. Bland–Altman analysis revealed no systematic bias, indicating robust geometric inference capability and providing reliable reference for quantitative assessment of pelvic floor support structures. However, dynamic parameters such as BND and ARJD showed slightly higher errors, mainly due to patient compliance during the Valsalva maneuver and the choice of temporal windows, suggesting that future optimization could involve standardized motion guidance and temporal modeling to enhance the accuracy and stability of dynamic measurements. These results demonstrate that the proposed model achieves both high segmentation accuracy, with a Dice coefficient of 88.67 ± 1.96%, and strong agreement with manual annotations, with a Pearson *r* up to 0.92, validating its reliability for clinical TPUS assessment.

In terms of clinical discriminative capability, significant differences (*p<* 0.05) were observed in dynamic parameters including BND, URA, V-BSD, and V-PUA between patients undergoing TAH and TLH. These parameters are internationally recognized indicators of pelvic floor support and mobility, and their variation directly reflects postoperative PFD status. These findings indicate that the automatically computed parameters can sensitively capture the functional alterations of pelvic floor support structures induced by different surgical approaches, serving as quantitative indicators for postoperative recovery assessment. This underscores the potential of TPUS-based automated analysis to facilitate individualized rehabilitation planning and risk prediction in clinical practice. Overall, the proposed multi-scale shifted window Transformer consistently outperforms CNN-based and hybrid Transformer models in segmentation and key anatomical point, providing clearer boundaries and better structural continuity for reliable TPUS analysis.

We would like to clarify that our patients were not normal in terms of pelvic floor function. Although they did not have PFD before the surgery, total hysterectomy itself is known to alter pelvic floor anatomy and function (2, 5). The functional parameters measured by our research group, such as BND and URA, fell within ranges consistent with mild to moderate pelvic floor impairment as reported in the literature. For instance, mean BND was 9.06 mm in the TAH group and 7.67 mm in the TLH group, values that exceed the normal threshold of approximately 5–6 mm suggested in previous studies (8, 13). Therefore, this group exhibited measurable changes related to PFD, and the high consistency between automatic measurement and manual measurement reflects the robustness of our method under clinical relevant conditions, rather than an accidental phenomenon in the normal group.

Despite these promising results, several limitations remain. First, the sample size was relatively small, and the postoperative follow-up period was short, limiting assessment of long-term stability of the model in rehabilitation monitoring. Second, modeling was based on two-dimensional TPUS images, which does not fully exploit volumetric information available from three- or four-dimensional ultrasound. Future work could integrate 3D-TPUS or shear wave elastography (SWE) to jointly assess pelvic floor muscle elasticity and structure. Additionally, the generalizability across centers, devices, and patient populations requires further validation to enhance model robustness and translational potential.

Moreover, this study was conducted using a single ultrasound system, namely Mindray RESONA R9T, at a single center with 50 patients who had benign gynecological conditions. The absence of external validation or testing across different ultrasound vendors and broader patient demographics inevitably limits real-world generalizability. To partially address this concern, we performed patient-level five-fold cross-validation, which yielded stable segmentation performance and indicated no severe overfitting to a specific test split. In addition, potential failure modes include severe pelvic organ prolapse, suboptimal Valsalva execution, and other atypical anatomical variations, which may lead to increased localization errors or measurement deviations. Domain shift is also a concern when extending the method to gynecologic oncology patients, whose anatomy may be more complex due to adjuvant therapies such as radiotherapy or surgery. To mitigate these risks, future work could incorporate multi-center datasets, transfer learning, or domain adaptation strategies, and implement patient-specific quality control measures during Valsalva maneuvers.

## 5 Conclusions

This work presents a multi-scale shifted window Transformer, enabling high-precision automated segmentation and key point detection of critical pelvic floor anatomical structures from TPUS images. Seven functional parameters were fully automatically measured via a geometric reasoning module. Experimental results demonstrate that the proposed method significantly outperforms conventional approaches in segmentation accuracy, key point localization, functional parameter consistency, and clinical discriminative capability. By enabling objective and reproducible evaluation, it provides a foundation for monitoring pelvic floor dysfunction and supporting postoperative rehabilitation after hysterectomy. This approach effectively replaces manual measurements, enhancing the efficiency and standardization of postoperative pelvic floor assessment. Although the current validation was performed in patients with benign disease, the methodology may be extended to gynecologic oncology populations with further validation, where the need for accurate, non-invasive assessment of PFD is even greater.

Therefore, future work will include collecting TPUS data from two additional centers using GE Voluson and Philips Affiniti systems, and we plan to evaluate the model on an independent cohort of at least 100 patients, including those with prior pelvic malignancy and severe anatomical distortion. Domain adaptation strategies will also be explored to enhance cross-device robustness.

## Data Availability

The original contributions presented in the study are included in the article/supplementary material. Further inquiries can be directed to the corresponding author.

## References

[1] Dai J Jin Z Zhang X Lian F Tu J . Efficacy of warm acupuncture therapy combined with kegel exercise on postpartum pelvic floor dysfunction in women. Int Urogynecology J. (2024) 35:599–608. doi: 10.1007/s00192-023-05698-9 38236284 PMC11023953

[2] Lai Y Lin A Zheng Z Wang Y Yu H Jiang X . Perceptions of pelvic floor dysfunction and rehabilitation care amongst women in southeast China after radical hysterectomy: a qualitative study. BMC Women’s Health. (2022) 22:108. doi: 10.1186/s12905-022-01687-0 35397542 PMC8994321

[3] Till S Schrepf A As-Sanie S . Impact of high tone pelvic floor dysfunction on change in sexual function following hysterectomy. Obstetrics Gynecology. (2025) 145:127S. doi: 10.1097/aog.0000000000005851.190 42160389

[4] Yaşar AB Yüzok RB Dağıstan E . Volumetric segmentation analysis of the levator ani muscle using magnetic resonance imaging in pelvic floor function assessment. Diagn Interventional Radiol. (2024) 30:220. doi: 10.4274/dir.2024.232586 PMC11589512 38375767

[5] Ji R He B Wu J . Application of transperineal ultrasound combined with shear wave elastography in pelvic floor function assessment after hysterectomy. Medicine. (2023) 102:e32611. doi: 10.1097/md.0000000000032611 36637923 PMC9839210

[6] Cai S Xia M Ding Y Zeng L . Clinical value of transperineal ultrasound in evaluating the effects of different delivery methods on the primipara pelvic floor structure and function. Sci Rep. (2024) 14:23980. doi: 10.1038/s41598-024-75014-y 39402151 PMC11473786

[7] Degirmenci Y Steetskamp J Schwab R Hasenburg A Schepers M Shehaj I . Functional assessment of anal sphincter with transperineal ultrasound and its relationship to anal continence. Diagnostics. (2024) 14:2614. doi: 10.3390/diagnostics14232614 39682523 PMC11640527

[8] Chen Y Peng L Liu M Shen H Luo D . Diagnostic value of transperineal ultrasound in patients with stress urinary incontinence (sui): a systematic review and meta-analysis. World J Urol. (2023) 41:687–93. doi: 10.1007/s00345-022-04264-0 36598556

[9] Guo B Stephans K Godley A Kolar M Magnelli A Tendulkar R . Transperineal ultrasound is a good alternative for intra-fraction motion monitoring for prostate stereotactic body radiotherapy. J Appl Clin Med Phys. (2023) 24:e14021. doi: 10.1002/acm2.14021 37144947 PMC10562017

[10] Sun S Li H Liu M Shang Q Tan Q Yin W . An evaluation of the effects of gestational weight gain on the early postpartum pelvic floor using transperineal ultrasound. J Ultrasound Med. (2023) 42:2331–8. doi: 10.1002/jum.16257 37255035

[11] Deng S Jiang Q Zhang Y . Application of transperineal pelvic floor ultrasound in postpartum pelvic organ injury and prolapse in women. Am J Trans Res. (2024) 16:4830. doi: 10.62347/hfeq7335 39398589 PMC11470310

[12] García-Mejido JA Ramos-Vega Z Fernández-Palacín A Borrero C Valdivia M Pelayo-Delgado I . Predictive model for the diagnosis of uterine prolapse based on transperineal ultrasound. Tomography. (2022) 8:1716–25. doi: 10.3390/tomography8040144 35894009 PMC9326672

[13] Wang J Yang X Wu Y Peng Y Zou Y Lu X . Deep learning–assisted two-dimensional transperineal ultrasound for analyzing bladder neck motion in women with stress urinary incontinence. Am J Obstetrics Gynecology. (2025) 232:e1.112. doi: 10.1016/j.ajog.2024.07.021 39032723

[14] van den Noort F Sirmacek B van der Vaart C Slump C . Deep learning enables automatic 3d segmentation of the puborectalis muscle on transperineal ultrasound. Int Urogynecology J. (2020) 31:S49–50.

[15] Joshi G Jain A Araveeti SR Adhikari S Garg H Bhandari M . Fda-approved artificial intelligence and machine learning (ai/ml)-enabled medical devices: an updated landscape. Electronics. (2024) 13:498. doi: 10.3390/electronics13030498 30654563

[16] Borys K Schmitt YA Nauta M Seifert C Krämer N Friedrich CM . Explainable ai in medical imaging: An overview for clinical practitioners–beyond saliency-based xai approaches. Eur J Radiol. (2023) 162:110786. doi: 10.1016/j.ejrad.2023.110787 36990051

[17] Zhang W Cai M Lee HJ Evans R Zhu C Ming C . Ai in medical education: Global situation, effects and challenges. Educ Inf Technol. (2024) 29:4611–33. doi: 10.1007/s10639-023-12009-8 30311153

[18] Li Q Qin Y . Ai in medical education: medical student perception, curriculum recommendations and design suggestions. BMC Med Educ. (2023) 23:852. doi: 10.1186/s12909-023-04700-8 37946176 PMC10637014

[19] Yousef R Khan S Gupta G Siddiqui T Albahlal BM Alajlan SA . U-net-based models towards optimal mr brain image segmentation. Diagnostics. (2023) 13:1624. doi: 10.3390/diagnostics13091624 37175015 PMC10178263

[20] Zhou Q Huang Z Ding M Zhang X . Medical image classification using light-weight cnn with spiking cortical model based attention module. IEEE J BioMed Health Inf. (2023) 27:1991–2002. doi: 10.1109/jbhi.2023.3241439 37022371

[21] Sefti R Sbibih D Jennane R . A cnn-based spline active surface method with an afterbalancing step for 3d medical image segmentation. Math Comput Simul. (2024) 225:607–18. doi: 10.1016/j.matcom.2024.06.002 38826717

[22] Fu B Peng Y He J Tian C Sun X Wang R . Hmsu-net: A hybrid multi-scale u-net based on a cnn and transformer for medical image segmentation. Comput Biol Med. (2024) 170:108013. doi: 10.1016/j.compbiomed.2024.108013 38271837

[23] Ao Y Shi W Ji B Miao Y He W Jiang Z . Ms-tcnet: An effective transformer–cnn combined network using multi-scale feature learning for 3d medical image segmentation. Comput Biol Med. (2024) 170:108057. doi: 10.1016/j.compbiomed.2024.108057 38301516

[24] Xia W Ameri G Fakim D Akhuanzada H Raza MZ Shobeiri SA . Automatic plane of minimal hiatal dimensions extraction from 3d female pelvic floor ultrasound. IEEE Trans Med Imaging. (2022) 41:3873–83. doi: 10.1109/TMI.2022.3199968 35984794

[25] Gao G Zhang H Xia Z Luo X Shi Y-Q . Reversible data hiding-based contrast enhancement with multi-group stretching for roi of medical image. IEEE Trans Multimedia. (2023) 26:3909–23. doi: 10.1109/tmm.2023.3318048 25079929

[26] Makhlouf A Maayah M AbuGhanam N Catal C . The use of generative adversarial networks in medical image augmentation. Neural Comput Appl. (2023) 35:24055–68. doi: 10.1007/s00521-023-09100-z 30311153

[27] Peng Y Meng Z Yang L . Image-to-image translation for data augmentation on multimodal medical images. IEICE Trans Inf Syst. (2023) 106:686–96. doi: 10.1587/transinf.2022dlp0008

[28] Ullah I Ali F Shah B El-Sappagh S Abuhmed T Park SH . A deep learning based dual encoder–decoder framework for anatomical structure segmentation in chest x-ray images. Sci Rep. (2023) 13:791. doi: 10.1038/s41598-023-27815-w 36646735 PMC9842654

[29] Pacal I Alaftekin M Zengul FD . Enhancing skin cancer diagnosis using swin transformer with hybrid shifted window-based multi-head self-attention and swiglu-based mlp. J Imaging Inf Med. (2024) 37:3174–92. doi: 10.1007/s10278-024-01140-8 38839675 PMC11612041

[30] Han Z Chen X Ye Z Su Y Wang L Mei S . Ustnet: A u-net swin transformer network for aerial visible-to-infrared image translation. IEEE Trans Geosci Remote Sens. (2025) 63:1–16. doi: 10.1109/tgrs.2025.3604474 25079929

[31] Qureshi JA Abdollahi G Tabib-Azar M . “ Atomic resolution mapping and electrochemical analysis of illicit drug-aptamer complexes and their terahertz signatures”, in: 2025 IEEE 20th Nanotechnology Materials and Devices Conference (NMDC) (Piscataway, NJ: IEEE), (2025) 305–9. doi: 10.1109/NMDC64551.2025.11234005

[32] Alinezhad E Mohammadzadeh AK Masoud S . Enhancing human-robot interaction through ensemble intention recognition and trajectory tracking. In: IISE Annual Conference Proceedings. (Norcross, GA: Institute of Industrial and Systems Engineers (IISE) (2025). p. 1–6.

[33] Guan Q Fang H Han C Wang Z Zhang R Zhang Y . Structural transformer with region strip attention for video object segmentation. Neurocomputing. (2024) 596:128076. doi: 10.1016/j.neucom.2024.128076 38826717

[34] Hu Z Zhang C Wang X Ge A . Light-adaptive human body key point detection algorithm based on multi-source information fusion. Sensors. (2024) 24:3021. doi: 10.3390/s24103021 38793877 PMC11125227

[35] Zhang Z Hu J Wang R Chen X Yang D Vavilov VP . Automatic segmentation of microporous defects in composite film materials based on the improved attention u-net module. Quantitative InfraRed Thermography J. (2025) 22:313–28. doi: 10.1080/17686733.2024.2387406 37339054

[36] Zhang W Ji D Yang W Zhao Q Yang L Zhuoma C . Application of swin-unet for pointer detection and automatic calculation of readings in pointer-type meters. Meas Sci Technol. (2023) 35:025904. doi: 10.1088/1361-6501/ad0c2f 30793291

[37] Zuo J Simpson DG O’Brien WD McFarlin BL Han A . Automated field of interest determination for quantitative ultrasound analyses of cervical tissues: Toward real-time clinical translation in spontaneous preterm birth risk assessment. Ultrasound Med Biol. (2024) 50:1861–7. doi: 10.1016/j.ultrasmedbio.2024.08.011 39271408 PMC11490401

